# Prevalence of bacterial vaginosis and factors associated among women who have sex with women^*^

**DOI:** 10.1590/1518-8345.2491.3077

**Published:** 2018-11-14

**Authors:** Mariana Alice de Oliveira Ignacio, Juliane Andrade, Ana Paula Freneda de Freitas, Gabriel Vitor da Silva Pinto, Marcia Guimarães da Silva, Marli Teresinha Cassamassimo Duarte

**Affiliations:** 1 Universidade Estadual Paulista, Faculdade de Medicina de Botucatu, Botucatu, SP, Brazil.; 2 Universidade de Brasília, Faculdade de Ciências da Saúde, Brasília, DF, Brazil.; 3 Universidade Estadual Paulista, Centro de Saúde Escola, Botucatu, SP, Brazil.

**Keywords:** Bacterial Vaginosis, Microbiota, Risk Factors, Prevalence, Female Homosexuality, Sexual and Reproductive Health

## Abstract

**Objective:**

to describe the prevalence of bacterial vaginosis and factors associated among women who have sex with women.

**Method:**

cross-sectional, descriptive and analytical study with 150 women. The vaginal microbiota profile was analyzed by microscopic examination of vaginal swabs according to the Gram method. Endocervical samples were collected with *cytobrush* for the investigation of endocervicitis by *Chlamydia trachomatis*. The polymerase chain reaction was used to diagnosis Human Papillomavirus infection. Socio-demographic data, sexual behavior and clinical history were obtained through an interview. Logistic regression was performed to identify risk factors independently associated with bacterial vaginosis.

**Results:**

among the 150 participants, 71 (47.3%) presented some alteration in the vaginal microbiota, 54 (36.0%) bacterial vaginosis and 12 (8.0%) Flora II. The variable independently associated with bacterial vaginosis was the use of sexual accessories [2.37(1.13-4.97), p=0.022].

**Conclusion:**

the high prevalence of bacterial vaginosis among women who have sex with women indicates the need for screening this population and association between use of sexual accessories and this disease suggests the possibility of transmission of sexual fluids between the partners during the sexual act, which demonstrates the need for educational actions on sexual and reproductive health.

## Introduction

A normal vaginal microbiota is highlighted in the literature as an important protective factor against genital tract pathogens^1^. One of the alterations in the vaginal microbiota is bacterial vaginosis (BV), characterized by reduction or depletion of lactobacilli that produce hydrogen and too much growth of anaerobic or facultative anaerobic microorganisms such as *Gardnerella vaginalis, Mobiluncus spp., Mycoplasma hominis, Prevotella sp., Porphyromonas spp.*, e *Peptostreptococcus spp.* ^2^ ^-^ ^3^. It is the most common cause of abnormal vaginal flora and a frequent reason women seek gynecological care^4^.

The importance of BV is not only related to its high prevalence in different populations, but also to associated obstetric and gynecological complications, including pelvic inflammatory disease^5^, premature labor^5^ and increased incidences of sexually transmitted infections (STI) such as cervicitis by *Chlamydia trachomatis* and *Neisseria gonorrhoeae* ^6^, as well as *Trichomonas vaginalis* ^7^ ^)^ and human immunodeficiency virus (HIV)^8^.

There are few studies in the world that addresses abnormal vaginal microbiota among women who have sex with women (WSW)^9^ and those that exist point to BV as the main alteration^10^ ^-^ ^12^. The number of female sexual partners was one of the main risk factors related to this condition among WSW^10^ ^-^ ^11^. In Brazil, only one article was published in 2005 on the subject and it found a high prevalence of BV among the WSW investigated ^12^.

The magnitude of BV and the gaps in the literature justify the present investigation, which aimed to describe the prevalence and factors associated with BV among WSW.

## Method

Cross-sectional, descriptive and analytical study part of a broader study aimed at assessing access to health services and sexual and reproductive health of WSW. The study was conducted in the city of Botucatu, São Paulo (SP), located in the middle of the state of São Paulo.

The study population consisted of women who reported having sex with women or with women and men, aged 18 years and older and who lived in the health micro-regions Pólo Cuesta, Vale do Jurumirim, Bauru and Jaú, part of the VI Regional Department of Health - Bauru.

The inclusion criteria were: being a woman and reporting having sex with women or with men and women and being 18 years old or older. Exclusion criteria were: not participating in all the steps proposed in the study - answering the questionnaire and performing a gynecological examination - and an inappropriate vaginal or endocervical sample for the laboratory tests.

To publicize the research in order to obtain the sample, a name and logo were created for the project, which was called: “Project Caring for the Health of Women that have Sex with Women”. A Facebook page was created (www.facebook.com/cuidandodasaudedamulher), in addition to the e-mail projetocsWSW@gmail.com, a poster and a brochure that were distributed in bars and clubs, Lesbians, Gays, Bisexuals and Transgender (LGBT) activist groups and health and teaching institutions. In addition to these means of communication, the project was also disseminated in radios, city newspapers, regional boards of managers, regional meetings of nurses, the campaign “*Fique Sabendo*”, Health Units in Botucatu, lectures, activities developed by community health workers of the Municipal STD/AIDS Program of Botucatu - SP and by the participating women themselves, who were asked to provide the telephone contact of other WSW of their community, applying the Snowball Sampling Technique^13^.

**Figure 1:**
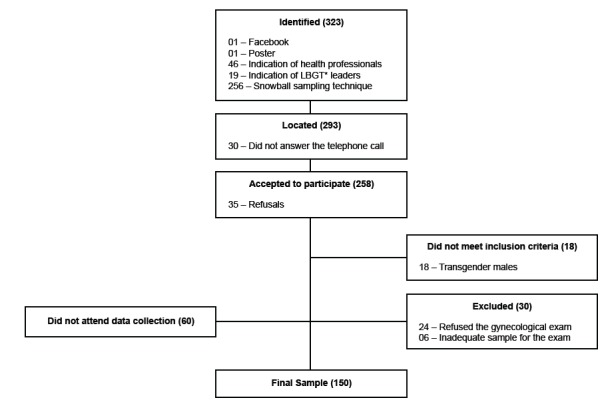
Diagram of sample selection

The researchers contacted the women indicated by health professionals, LGBT leaders or research participants and invited them to participate, clarifying the objectives, form of participation in the study, and scheduling dates and times. Thus, 323 WSW were identified and 293 of them were contacted, since 30 women were not located after three telephone calls at different days and times. Of these, 35 refused to participate in the study and 18 did not meet the inclusion criteria, totaling a sample of 240 women. Of these, 60 did not attend data collection after scheduling it three times and 30 were excluded (24 did not accept the gynecological examination and the cervical sample of six women was inadequate for the laboratory diagnosis of Chlamydia trachomatis and human papillomavirus - HPV). The final sample consisted of 150 WSW, and the detail of its constitution is explained in the diagram below (Figure 1).

*LGBT- Lesbian, Gay, Bisexual, and Transgender

The outcome variable was bacterial vaginosis (yes/no) and the independent variables analyzed are described below. The sociodemographic variables were: age in years (<19, 20-29, 30-39, 40-49, ≥50), skin color (white/non-white), civil status (married/stable union) and years of education. Regarding substance use, behavior and sexual practices the variables were: tobacco use (yes/no), number of male sexual partners 3 months, number of female sexual partners 3 months, casual partners 3 months (yes/no), fixed partner (yes/no), use of vaginal douche (yes/no), vaginal penetration (yes/no), use of sexual accessories (yes/no), shares sexual accessories (yes/no), condom use - considered for all anal and vaginal sex (yes/no), anal penetration (yes/no). The clinical variables were: hormonal contraceptive (yes/no), Chlamydia trachomatis infection (yes/no) and HPV infection (yes/no).

Data were collected by the authors from January 2015 to April 2017 through the application of a questionnaire that addressed the variables listed above and the gynecological examination. During the examination, vaginal swabs were collected for analysis of the vaginal microbiota by microscopic examination according to the Gram method^14^. Vaginal candidiasis was diagnosed by visualization of blastoconidia and/or pseudohyphae. Diagnosis of HPV infection and Chlamydia trachomatis were obtained by polymerase chain reaction (PCR). All the tests were performed at the Maternal-Fetal Relationship Immunopathology Laboratory, Pathology Department, Botucatu Medical School (FMB), Universidade Estadual Paulista (UNESP).

The data was analyzed by descriptive statistics and the associations between the independent variables and diagnosis of BV were calculated using a simple logistic regression model. The variables that most influenced the outcome (p<0.20) were analyzed in a model of multiple logistic regression to identify those independently associated with the outcome (p<0.05). SPSS 21.0 software was used for the analysis.

The research project was approved by the Research Ethics Committee of FMB-UNESP, protocol number 837.447 and it complies with all standards for research involving human beings. After clarifying the study, the women were invited to participate and those who agreed signed an Informed Consent Term. All women with positive results were referred for treatment and follow-up.

## Results

Among the 150 WSW included in the study, those in the age group of 20 to 49 years (83.3%), white (74.7%), single (73.3%) and with 12 or more years of education (51.3%) predominated. Most of them were smokers (43.3%). Regarding sexual practices, the majority did not have sex with men in the last three months (88.0%), had only one sexual partner in this period (82.0%) and had vaginal penetration (88.0%) and oral sex (96.0%). Almost one third (31.3%) used accessories in sexual practices and 21.3% shared them. Consistent use of condoms in anal and vaginal intercourse was reported by only 18.0% of the women included in the study and 20.0% used vaginal douches (Table 1). All women did not use condoms in oral sex.

**Table 1:** Socio-demographic variables and variables related to substance use, behavior and sexual practices of women who have sex with women. Botucatu, SP, Brazil, 2015-2017

Variables	n	%
Age		
≤ 19	16	10.7
20 -29	79	52.6
30-39	33	22.0
40-49	13	8.7
≥ 50	09	6.0
Skin color		
White	112	74.7
Non-White	38	25.3
Marital Status		
Married/stable union	40	26.7
Single	110	73.3
Years of education		
5 -7	06	4.0
8 -11	67	44.7
≥ 12	77	51.3
Use of Tobacco		
Yes	65	43.3
No	85	56.7
No. of male partners 3 months		
0	132	88.0
1	14	9.3
≥2	04	2.7
No. of female partners 3 months		
0	15	10.0
1	123	82.0
≥2	12	8.0
Vaginal penetration	139	92.6
Anal penetration	25	16.6
Oral sex	144	96.0
Uses sexual accessories	47	31.3
Shares sexual accessories	32	21.3
Condom use*		
Yes	27	18.0
No	123	82.0
Vaginal douche	30	20.0

* Condom use - in all anal and vaginal sexual practices

The vaginal microbiota profiles of the women included in the study is presented in Table 2. It was observed that almost half (47.3%) of the WSW investigated presented some alteration of the vaginal microbiota, with BV being the most prevalent (36.0%), followed by Flora II (8.0%). Vaginal candidiasis was detected in four women (2.7%) (Table 2).

**Table 2:** Vaginal microbiota profiles of women who have sex with women (n=150). Botucatu, SP, Brazil, 2015-2017

Vaginal Microbiota	n	%
Flora I	75	50.0
Bacterial Vaginosis	54	36.0
Flora II	12	8.0
Flora I + Vaginal candidiasis	04	2.7
Other alterations in microbiota*	05	3.3
Total	150	100.0

* Other changes in microbiota - cocci and abnormal flora

The associations between BV and socio-demographic variables and those related to substance use, sexual and clinical practices and behaviors are presented in Table 3.

**Table 3:** Association between bacterial vaginosis and sociodemographic variables and variables related to substance use, behavior, sexual and clinical and practices. Botucatu, SP, Brazil, 2015 - 2017

Variables	Vaginose Bacteriana				OR*(95%CI^†^)			*P* ^‡^
	Total	%	No (n=96)		Yes (n=54)			
			n	%	N	%		
Age								
18-19	16	10.6	12	75.0	04	25.0	1	
20-29	79	52.6	49	62.0	30	38.0	1.84(0.54-6.22)	0.328
30-39	33	22.0	19	57.6	14	42.4	2.21(0.59-8.32)	0.241
40-49	13	8.6	09	69.2	04	30.7	1.33(0.26-6.83)	0.730
≥50	09	6.0	07	77.7	02	22.2	0.86(0.12-5.94)	0.876
Color								
White	112	74.6	73	65.1	39	34.8	1	
Non-White	38	25.3	23	60.5	15	39.5	1.22(0.57-2.60)	0.606
Marital Status								
Married/Stable union	40	26.6	26	65.0	14	35.0	1	
Single	110	73.3	70	63.6	40	36.3	1.06(0.50-2.26)	0.878
Years of education							0.97(0.86-1.09)	0.583
Tobacco use								
No	85	56.6	59	69.4	26	30.6	1	
Yes	65	43.3	37	56.9	28	43.0	1.72(0.88-3.37)	0.116
No. of partners 3 months							1.10(0.54-2.25)	0.789
No. of partners 3 months							0.91(0.58-1.42)	0.673
Casual partners 3 months								
No	118	78.6	75	63.5	43	36.4	1	
Yes	32	21.3	21	65.6	11	34.3	0.91(0.40-2.07)	0.829
Fixed partner								
No	33	22.0	21	63.6	12	36.3	1.02(0.45-2.27)	0.961
Yes	117	78.0	75	64.1	42	35.8	1	
Vaginal douche								
No	120	80.0	79	65.8	41	34.1	1	
Yes	30	20.0	17	56.6	13	43.3	1.47(0.65-3.33)	0.351
Vaginal penetration								
No	11	7.3	09	81.8	02	18.2	1	
Yes	139	92.6	87	62.5	52	37.4	2.69(0.56-12.93)	0.217
Sexual accessories								
No	103	68.6	73	70.8	30	29.1	1	
Yes	47	31.3	23	48.9	24	51.0	2.53(1.25-5.18)	0.010
Condom use								
No	123	82.0	77	62.6	46	37.3	1.41(0.57-3.50)	0.448
Yes	27	18.0	19	70.3	08	29.6	1	
Anal penetration								
No	125	83.3	81	64.8	44	35.2	1	
Yes	25	16.6	15	60.0	10	40.0	1.22(0.51-2.96)	0.648
Oral sex								
No	06	4.0	05	83.3	01	16.6	1	
Yes	144	96.0	91	63.2	53	36.8	2.91(0.33-25.60)	0.335
Hormonal contraceptive								
No	124	82.6	76	61.3	48	38.7	2.10(0.78-5.61)	0.137
Yes	26	17.3	20	76.9	06	23.0	1	
*C.T* ^§^ infection								
No	147	98.0	94	64.0	53	36.0	1	
Yes	03	2.0	02	66.6	01	33.3	0.88(0.07-10.01)	0.923
HPV^||^ infection								
No	82	54.6	57	69.5	25	30.4	1	
Yes	68	45.3	39	57.3	29	42.6	1.69(0.86-3.32)	0.124

*OR- odds ratio; †CI- confidence interval; ‡*P-*p-value; §*C.T*- *Chlamydia trachomatis;* ||HPV- human papillomavirus

The variables that were most associated with BV in the simple logistic regression were: tobacco use [1.72(0.88-3.37), p=0.116], use of sexual accessories [2.53(1.25-5.18), p=0.010], hormonal contraceptive [2.10(0.78-5.61), p=0.137] and HPV infection [1.69(0.86-3.32), p=0.124] (Table 3).

In the multivariate analysis, only the variable use of sexual accessories was independently associated with BV. Women who used sexual accessories had a two and a half greater chance of having a positive diagnosis of BV than those who did not [2.37(1.13-4.97), p=0.022] (Table 4).

**Table 4:** Multivariate analysis of risk variables for bacterial vaginosis. Botucatu, SP, Brazil, 2015 - 2017

Variables	ORa* (IC†95%)	P^‡^
Tobacco use		
No	1	
Yes	1.68(0.83-3.40)	0.147
Uses sexual accessories		
No	1	
Yes	2.37(1.13-4.97)	0.022
Hormonal contraceptive		
No	2.39(0.86-6.62)	
Yes	1	0.093
HPV^§^ Infection		
No	1	
Yes	1.57(0.78-3.17)	0.210

*ORa - adjusted odds ratio; †IC- confidence interval; ‡*P-*p-value; §HPV - papilloma human virus

## Discussion

The present study, aimed at assessing the prevalence of BV and associated factors in a WSW sample, identified as high prevalence of this disease and the use of sexual accessories as an independently associated variable.

Among the alterations in vaginal microbiota, BV was the most prevalent. The overall prevalence of alterations in vaginal microbiota obtained in the present study was higher than that obtained in a study conducted in the United States of America (USA) with WSW (47.3% vs 36.0%) and similar to that obtained in another American study that investigated African-American WSW (47.5%). Both studies evaluated the vaginal microbiota profile using the same criteria used in the present investigation^14^.

The prevalence of BV among the WSW included in this study (36.0%) was higher than the prevalence found in American^11^ ^,^ ^15^ ^-^ ^16^ and Australian^17^ studies with WSW, which also used the same criteria of this investigation^14^ for diagnosis and found results ranging from 25.0% to 28.7%. However, this result was lower than the results of American studies conducted in 2013^18^ and 2018^19^ with WSW, which found a prevalence of 40.3% and 56.0%, respectively. This difference can be justified by the sample, since the American studies^19^ ^-^ ^20^ ^)^ investigated African-American women. Women of color have already been highlighted as a factor associated with BV in a previous study^20^.

An English study^10^ conducted in a clinic specialized in sexual health care for lesbians and bisexuals showed a 31.4% prevalence of BV and a national research^12^ conducted with WSW in the city of São Paulo found a 33.8% prevalence of BV. Both studies found values close to that found in the present investigation, despite using different diagnostic criteria^21^ that are not considered gold standard for the diagnosis of BV.

Thus, the biological vulnerability of the women investigated is considered high, since BV is a disease significantly associated with the acquisition of STI/HIV^6^ ^,^ ^8^.

The present investigation found an association between the use of sexual accessories and BV. Previous studies have already pointed out that the use of these objects is associated with BV in WSW^11^ ^,^ ^22^. Therefore, the findings of the present investigation corroborate the hypothesis that BV is associated with sexual practices that transfer vaginal fluids between the partners^22^. However, there is still a need for research to deepen the knowledge about this interaction^22^ and educational actions on the use of condoms and hygiene of sexual accessories should be carried out.

An international study evaluating the factors associated with BV in WSW in England^10^ showed that a greater number of female sexual partners, race and smoking increased the risk of this outcome among the women participating in the study. A study conducted in the USA^11^ with WSW also found that the increase in the number of sexual partners was a factor independently associated with BV and that those receiving oral and anal sex were more likely to have BV. A literature review^9^ conducted in the USA with the objective of finding factors associated with BV in WSW indicated as associated factors the number of sexual partners, positive diagnosis of sexual partners, smoking and period of the menstrual cycle. These studies differ from the findings of the present investigation, which found no association with socio-demographic variables, substance use, clinical and behavioral variables, except for the use of sexual accessories.

A limitation of this study was the fact that it was conducted at the regional level and with a non-randomized sample. However, it is important to highlight the difficulty of finding the target population, which was previously demonstrated. In addition, the importance of the study must be noted, considering that a literature review^9^ conducted in 2015 found a small number of studies related to this theme and population group in the world.

This research contributes to increase the knowledge in the area, since it addresses an important issue for the health of this group. It also contributes to the practice of health professionals, since its findings suggest the need for an individualized professional approach, focused on sexual and reproductive health, based on preventive, diagnostic and therapeutic actions, with a view to comprehensive care.

## Conclusion

The high prevalence of BV among WSW indicates the need for screening this population. The association between use of sexual accessories and BV suggests the possibility of transmission of sexual fluids between the partners during the sexual act, which demonstrates the need for educational actions on sexual and reproductive health.
